# A one-man bilingual cocktail party: linguistic and non-linguistic effects on bilinguals’ speech recognition in Mandarin and English

**DOI:** 10.1186/s41235-024-00562-w

**Published:** 2024-06-05

**Authors:** Erin D. Smith, Lori L. Holt, Frederic Dick

**Affiliations:** 1 Department of Psychology, Carnegie Mellon University, Pittsburgh, USA; 2 College of Liberal Arts, Department of Psychology, The University of Texas at Austin, Sarah M. & Charles E. Seay Building, 108 E Dean Keeton St, Austin, TX 78712 USA; 3 Experimental Psychology, University College London, London, United Kingdom

**Keywords:** Speech recognition, Informational masking, Bilingual, Selective attention, Coordinate response measure

## Abstract

Multilingual speakers can find speech recognition in everyday environments like restaurants and open-plan offices particularly challenging. In a world where speaking multiple languages is increasingly common, effective clinical and educational interventions will require a better understanding of how factors like multilingual contexts and listeners’ language proficiency interact with adverse listening environments. For example, word and phrase recognition is facilitated when competing voices speak different languages. Is this due to a “release from masking” from lower-level acoustic differences between languages and talkers, or higher-level cognitive and linguistic factors? To address this question, we created a “one-man bilingual cocktail party” selective attention task using English and Mandarin speech from one bilingual talker to reduce low-level acoustic cues. In Experiment 1, 58 listeners more accurately recognized English targets when distracting speech was Mandarin compared to English. Bilingual Mandarin–English listeners experienced significantly more interference and intrusions from the Mandarin distractor than did English listeners, exacerbated by challenging target-to-masker ratios. In Experiment 2, 29 Mandarin–English bilingual listeners exhibited linguistic release from masking in both languages. Bilinguals experienced greater release from masking when attending to English, confirming an influence of linguistic knowledge on the “cocktail party” paradigm that is separate from primarily energetic masking effects. Effects of higher-order language processing and expertise emerge only in the most demanding target-to-masker contexts. The “one-man bilingual cocktail party” establishes a useful tool for future investigations and characterization of communication challenges in the large and growing worldwide community of Mandarin–English bilinguals.

## Significance Statement

Just five percent of the world’s estimated 7.5 billion English speakers are native; most speakers learn English as a second or later language. Yet, the role of multilingual speech processing in language and communication disorders, or even in healthy everyday listening, is not well understood. Advancing understanding will be important, as healthy multilingual listeners are known to find speech recognition challenging in noisy everyday contexts like cafés, classrooms, and parties. Appropriate and effective clinical and educational interventions will require understanding how language proficiency might interact with the degree and nature of a listening challenge. Here, we create a scenario in which the two most spoken languages on Earth—English and Mandarin—vie for listeners’ attention. Across two experiments we examine a “one-man cocktail party” in which the speech of one highly proficient Mandarin–English bilingual male competes across simultaneous presentation. This reduces low-level acoustic cues typically conflated with language in two-talker studies. Further, for both practical and theoretical purposes our task involves no language- or culturally specific knowledge. Holding voice constant across the speech, the results reveal that effects of higher-order language processing and expertise emerge only in the most demanding contexts. This advances understanding of why speech recognition is better across competing talkers speaking different languages and establishes a tool both for further investigation and characterization of communication challenges in the large and growing worldwide population of Mandarin–English bilinguals.

## Introduction

As anyone in a shared office, restaurant, or call center can attest, speech recognition is more challenging in the context of a competing talker. The so-called masking produced by another voice is traditionally categorized into two types: energetic and informational masking (Durlach et al., 2003). Energetic masking is defined as arising from acoustic overlap between an attended or “target” talker, and a masking “distractor” talker that drives competition between target and distractor encoding at the auditory periphery (Kidd et al., 2008; Pollack, 1975; Durlach et al., 2003). Informational masking is broadly considered to arise from interfering effects that are not accounted for by energetic masking (Leek et al., 1991).

Most of what we know about informational masking has come from studies solely focused on English (Wang & Xu, 2021). Yet, complex listening environments are increasingly linguistically heterogeneous. To take just one example, tens of millions of individuals worldwide speak both English and Mandarin, often with different levels of fluency (Ethnologue, 2022). The ever-growing population of Mandarin–English bilinguals will be listening and conversing in challenging multitalker situations in which conversational partners and competing talkers may be speaking in their dominant or non-dominant language—introducing a bilingual cocktail party challenge (Cherry, 1953).

These multitalker environments can be particularly hard for non-native speakers whose proficiency differs across native and non-native languages (Cooke et al., 2008; Morini & Newman, 2020), as well as older participants, and those with hearing or language impairments (Reiss & Molis, 2021; Ruggles et al., 2012). The combination of these factors has the potential to make already-challenging situations in daily life yet more difficult (for an analogous example of multifactorial effects in speech recognition, see Koeritzer et al., 2018). Understanding how language proficiency as well as interference from different languages might affect individual listeners is also vital for applied and clinical situations (Shi, 2014; Phillips et al., 2023).

Research that has examined informational masking in the context of one or more simultaneously presented languages introduces the term *linguistic release from masking* to describe an observed boost in speech recognition when the target and competing talker(s) speak different languages (Viswanathan et al., 2016), an influence that has been observed across many language combinations (Brouwer et al., 2012; Calandruccio et al., 2016; Cooke et al., 2008; Freyman et al., 2001; Rhebergen et al., 2005; Van Engen & Bradlow, 2007; Tun et al., 2002).

When a listener experiences linguistic release from masking, it may be due to greater ease of filtering a non-intelligible distractor language, an increased ability to focus on the target speech stream, or both. Van Engen (2012) explored the relative weight of these abilities during speech-in-speech recognition through an auditory training study in which monolingual English speakers were presented with English-target sentences in speech-shaped noise (SSN), Mandarin babble, or English babble. Only listeners training in the babble conditions showed within-practice gains in speech recognition, indicating that listener experience may hold greater influence over informational masking than pure energetic masking. Additionally, listeners in all conditions demonstrated stronger speech recognition outcomes when the target sentences were spoken by a familiar talker. Strikingly, gains in English-target speech recognition were evident only for conditions in which the listener was trained, such that listeners trained in Mandarin babble did not show improvements in speech recognition outcomes during a posttest in English babble, and vice versa. Van Engen (2012) concluded that if listeners have access to the linguistic content of a masker, they may be able to more effectively separate it from the intended target speech stream (i.e., “tune out” the distractor) while also capitalizing on target talker familiarity (i.e., “tune in” to the target).

There remain open questions about the extent to which listeners’ knowledge of the competing language impacts linguistic release from masking. Calandruccio and Zhou (2014) report that English monolinguals and English-Greek bilinguals experience a nearly equivalent release from masking when listening to English targets in the context of competing Greek, despite the differences in Greek comprehension across groups. Further, studies have demonstrated that bilingual listeners can benefit from a target–distractor language mismatch, regardless of language proficiency (Brouwer et al., 2012; Van Engen, 2010). Yet, other studies suggest that language proficiency *can* play a role (for a recent review and data, see Phillips et al., 2023): Native language can be a more potent masker of a second-language (L2) target (Cooke et al., 2008; Garcia Lecumberri & Cooke, 2006). In general, when multiple speech streams vie for attention, recognition benefits from the presence of multiple languages. Yet, factors including language proficiency in each language, cognitive control abilities, linguistic complexity of the study materials, and the match between target and masking languages appear likely to exert interacting influences on listening in multilingual contexts (Filippi et al., 2012).

Similarly, there are questions about the extent to which low-level commonalities between target and masker languages drive informational masking in multi-language listening contexts. Previous research often has examined informational masking across Indo-European language pairs (Brouwer, 2017; Brouwer et al., 2012; Filippi et al., 2012), which are likely to share more acoustic similarity than more linguistically distant languages. Indeed, a distracting voice speaking a language phonetically similar to the target language (e.g., Dutch-English) produces greater masking than a phonetically dissimilar language (e.g., Mandarin–English; Brouwer et al., 2012; Calandruccio et al., 2013). This suggests a role for lower-level interference across acoustic–phonetic features of speech (Brouwer et al., 2012; Calandruccio et al., 2018). Yet, a recent well-powered study reporting linguistic release from masking found no corresponding evidence that acoustically dissimilar languages produce greater linguistic release from masking (Brown et al., 2022). The extent to which low-level commonalities between target and masker languages drive informational masking in multi-language listening contexts remains unclear, driven at least in part by studies’ differences in the number and types of maskers, vocal characteristics of the speakers, target–distractor language similarities, and differences in task structure.

Here, we examine Mandarin and English, the world’s two most spoken languages (Ethnologue, 2022), because of their substantial linguistic differences. Mandarin is a tonal language, in which lexical meaning is signaled by the contour of fundamental frequency (F0). It possesses acoustic–phonetic patterns well differentiated from English, as well as a distinctive rhythm and pause-to-speech ratios (Bradlow et al., 2011; Keating & Kuo, 2012). Differences like these may support a release from informational masking (Binns & Culling, 2007; Carlile & Corkhill, 2015). We take this one step further by creating a “one-man bilingual cocktail party” in which target and distractor speech streams are spoken by one male bilingual Mandarin–English speaker who is fluent to the point of being perceived as a native speaker in both languages. This (admittedly artificial, but see Driver, 1996) listening scenario permits us to examine linguistic release from masking in a context that eliminates as many talker-idiosyncratic vocal cues as possible, while retaining language-specific features. Finally, we rely on the Coordinate Response Measure (CRM) task (Bolia et al., 2000; Brungart, 2001), a widely used paradigm in the field of speech and hearing that requires the listener’s attention to a target speech stream in competition with a simultaneous and temporally aligned distractor speech phrase, presented diotically via headphones.

This approach confers several advantages. Low-level acoustic characteristics of the voice are well matched as there is a common talker even as Mandarin and English convey substantial language-relevant differences. Moreover, owing to the CRM task structure, syntactic and semantic predictability is eliminated, and the temporal evolution of the phrases is matched in time, thereby discouraging “glimpsing” of acoustic information from one talker when the other is quiet. The basic nouns inherent to the CRM require no language- or culturally specific knowledge, making them appropriate for use in a task with bilinguals of varying proficiency. Further, use of a small, closed set of basic colors and numbers minimizes cross-language vocabulary differences, as it is likely that second-language learners have been introduced and exposed to color/number nouns earlier and more repetitively than other nouns. Additionally, a closed-set speech corpus lowers the chance of errors in which the listener perceives a word not present in the bank of options, a source of comprehension error often present in informational masking studies (Cooke et al., 2008). In sum, we assess the potential for differences in listeners’ susceptibility to interference in conditions with low linguistic but high selective attention demands depending on the languages accessible to them.

In Experiment 1, we use the new single-talker Mandarin–English version of the CRM to provide multiple estimates of informational masking with minimal talker cues. Speech recognition accuracy differences with Mandarin versus English masking speech—spoken by the same talker—provides one estimate (linguistic release from masking). Another estimate is provided by the potential accuracy difference between bilinguals who understand both Mandarin and English versus non-Mandarin-speaking participants (henceforth referred to as English speakers for simplicity) when the competing speech is Mandarin. We also ask how speech recognition might be mediated by Mandarin–English bilinguals’ English proficiency. Finally, we ask to what degree recognition errors can be accounted for by intrusions from the masking talker.

## Experiment 1

Here, we recruited English listeners without Mandarin experience and bilingual Mandarin–English listeners to complete a CRM task where the target was presented in English and the distractor was presented in either English or Mandarin, with both sentences spoken by the same male talker. To reflect the real-world demands of varying levels of target and distractor voices in conversation, five levels of target-to-masker ratio (TMR) were employed, where the target speech was presented simultaneously with higher- or lower-amplitude distractors (positive and negative TMR, respectively). This protocol is in keeping with established CRM approaches to selection of TMR levels (e.g., Brungart et al., 2001; Brungart & Simpson, 2002; Iyer et al., 2010).

## Participants

The website Prolific (https://www.prolific.com/.) facilitated recruitment of 60 participants for online testing. We restricted recruitment to “native English” and “native Mandarin, proficient in English” participants using the Prolific recruitment screening tools that rely on participants’ intake surveys in registering to be participants through the website. By this approach, 30 participants (13 female, 1 preferring not to answer, ages 18–35 years inclusive) self-reported native familiarity with English. The remaining 30 bilingual Mandarin–English participants self-reported Mandarin as a first language (15 female, 1 non-binary, ages 18–35 years inclusive). For the purposes of data analysis, we used the language data reported to Prolific in the participant intake survey. Additionally, we asked our own demographic language questions in a separate survey linked to our study (see Appendix).

Two “native English” participants self-reported familiarity with Mandarin or Cantonese during a brief language questionnaire at the end of the experiment and were subsequently removed from analysis.

All participants had self-reported normal hearing. Online participants provided informed consent and received monetary compensation. The experiment procedures were approved by the Carnegie Mellon University Institutional Review Board.

### Stimuli

Stimuli were modeled after the CRM corpus (Bolia et al., 2000). Two phrases were presented diotically on each trial. Each stimulus was a pair of phrases following the structure of “Ready < call sign > , go to < color >  < number > now.” On each trial, one of the phrases contained the call sign “Baron,” to indicate the target talker participants should attend to. The “distractor” call signs and the colors and numbers of each phrase varied by trial. In this manner, the target phrase indicated by “Baron” was always in English, whereas the distractor phrase was in English or Mandarin, depending on the block and counterbalanced in order across participants. Table 1 shows the full list of stimulus attributes.

**Table 1:** Coordinate Response Measure (CRM) Attributes for Experiments 1 and 2

Keyword	English	Mandarin
Call Sign	Arrow, **Baron**, Charlie, Hopper, Eagle, Laker, Ringo, Tiger	教师 [jiao shi], 太阳 [tai yang], **天空 [tian kong],** 希望 [xi wang], 国家 [guo jia], 人们 [ren men], 学校 [xue xiao], 电视 [dian shi]
Color	Blue, Green, Red, White	蓝 [lan], 绿 [lv], 红 [hong], 白 [bai]
Number	1, 2, 3, 4, 5, 6	一, 二, 三, 四, 五, 六

Keywords used to generate the CRM speech stimuli. Each phrase followed the structure of *Ready* < *call sign* > *go to* < *color* >  < *number* > *now.* Call signs “Baron” and “天空 [tian kong]” were used to identify targets across Experiments 1 and 2 and are highlighted in bold

Both English and Mandarin stimuli were recorded by a bilingual native Mandarin speaker with high verbal fluency in both languages and minimal foreign accent using an Electro-Voice RE20 microphone (44.1 kHz) in a sound-attenuated booth. The use of the same voice for all speech stimuli, regardless of language, was intended to minimize identifiable low-level acoustic cues within the target and distractor speech, and across languages. One characteristic that might be expected to vary across Mandarin and English even within the same talker is the mean and variability of F0 (Keating & Kuo, 2012). Average and standard deviation F0 values were measured in Praat (Boersma & Weenink, 2021) for each set of recordings. For English, the overall mean F0 was 207.65(± 41.38) Hz; for Mandarin mean F0 was 188.27(± 57.90) Hz, a ~ 19 Hz (1.75 semitone) average difference. The CRM task provides a context in which speech cues are delivered with closely aligned spectrotemporal properties, reducing the utility of differences in F0 between the target and distractor speech cues and “glimpsing” of the target speech. However, at least in natural speech contexts, this mean F0 difference would likely support some release from masking (Deroche & Culling, 2013).

The audio recordings, sampled at 44.1 kHz, were RMS matched in MATLAB R2010a (The MathWorks, Inc.). Each recording was adjusted to match the mean duration of all utterances (2.108 s) using the Stretch and Pitch effect in Adobe Audition (version 13.0.13.46). The mean change in duration was 11.49% for all audio recordings, including those later mentioned in Experiment 2. Phrases were combined to form diotic, rhythmically similar phrase pairs in MATLAB with 10 ms zero padding at the beginning and end of each file, along with 10 ms cosine onset and offset amplitude ramps. Similar to Johnsrude et al. (2013), each target–distractor pair was amplitude-adjusted to create five TMRs, with the target being − 6 dB, − 3 dB, + 3 dB, + 6 dB, or equal in amplitude relative to the masker. Finally, all stimuli were amplitude normalized at 80% maximum in Adobe Audition to ensure that the perceptual loudness of the stimuli was consistent after adjustment for TMR level.

In the case of the 0 dB TMR stimulus presentation, the combined word pairs with English distractors were primarily differentiable based on suprasegmental cues such as prosody. Slightly different F0 values across individual stimuli may have also supported differentiation, especially in the case of trials with mismatching target and distractor languages. When the target and distractor cues were presented at different TMR levels, the difference in intensity served as an additional cue for tracking speech signals across the stimulus presentation.

**Fig. 1:**
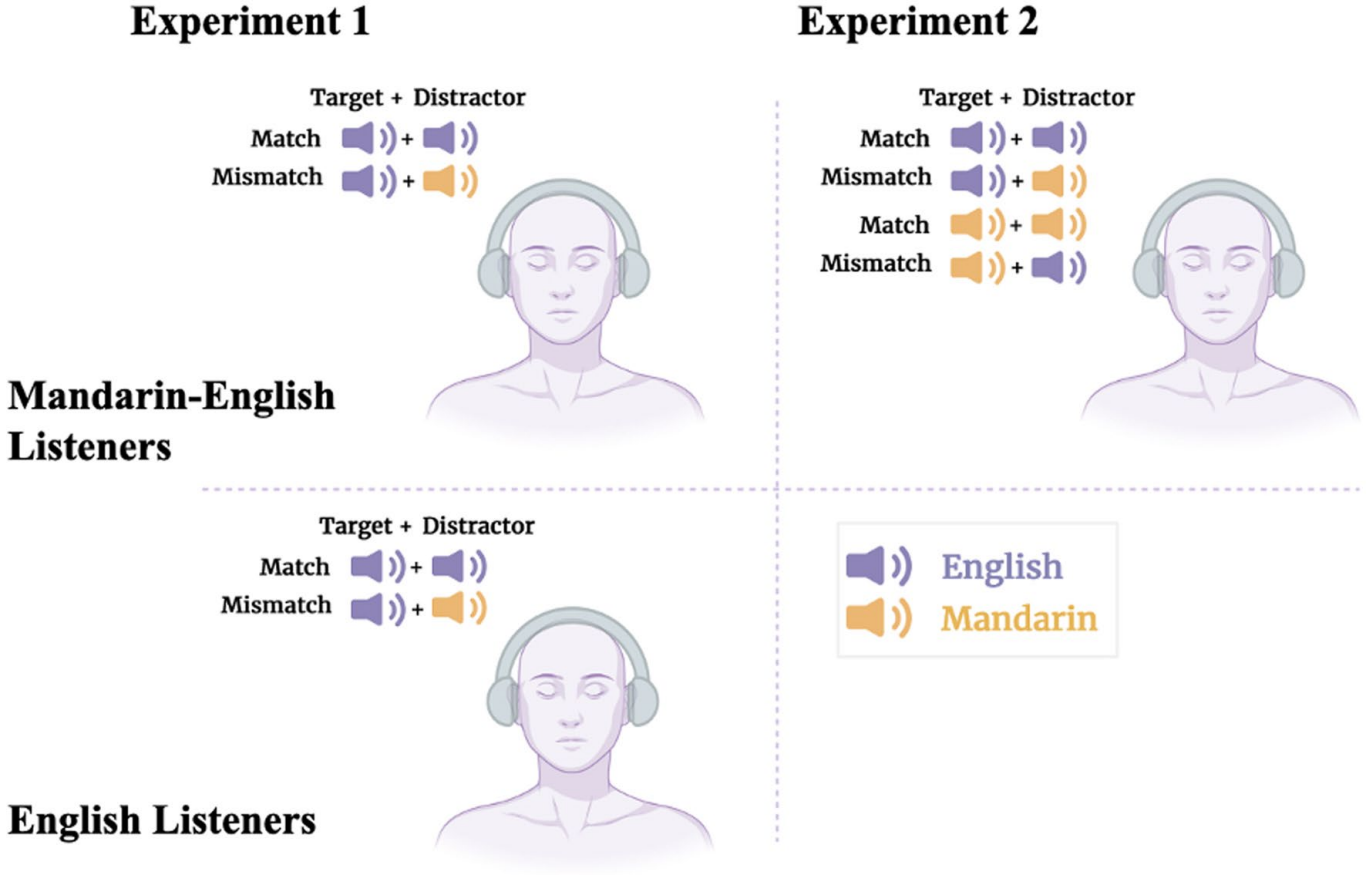
Task Schematic for Experiments 1 and 2. In each experiment, bilingual Mandarin–English or non-Mandarin-speaking English participants heard two competing speech streams over the left and right channels of their headphones. These streams either matched or mismatched in the language (English/Mandarin). Each speech stream followed a stylized pattern corresponding to “Ready <call sign> go to <color> <number> now.” Participants listened for their call sign (e.g., “Ready, Baron!”) to identify the target stream and report the color and number it conveyed on a 6 (number) × 4 (color) response grid. In Experiment 1, both bilingual Mandarin–English and non-Mandarin-speaking English participants responded to English language targets with distracting English and Mandarin speech across two task conditions. In Experiment 2, bilingual Mandarin–English participants responded to all combinations of Mandarin and English speech assigned as target versus distractor across four task conditions

As shown in Fig. 1, the target phrase was always English in Experiment 1 and it was indicated by the target call sign “Baron,” paired with each color/number combination. These English phrases were paired with an English- or Mandarin-distractor phrase, which was composed of a call sign, color, and number that always differed from the target phrase.

To prevent potential confusion among bilingual listeners, Mandarin call signs consisted of entirely new nouns, rather than translations of the English call signs. All call signs were bisyllabic regardless of language. Additionally, it was ensured that colors presented within the Mandarin speech phrases were always incongruous with their English speech phrase counterparts. For example, the Mandarin word for “red,” 红 [hong], was never presented on the same trial as an English phrase using the word “red.” 120 unique stimuli were generated per Mandarin/English condition, leading to 240 audio files total. Phrase pairs were randomly assigned to a TMR, such that each TMR was represented by 48 trials, or 24 trials per condition.

This created a pool of stimuli expected to place heavy auditory and informational selective attention demands on English and bilingual Mandarin–English participants. Both groups possessed sufficient English language expertise to comprehend the simple target phrases. The English distractors were likewise intelligible to both groups, but English proficiency among Mandarin–English bilinguals may impact recognition under these demanding selective attention conditions. In contrast, Mandarin distractors are intelligible to bilingual participants but unintelligible to participants in the English group, selected to have no knowledge of Mandarin.

## Procedure

The online experiment was completed in a single session via Gorilla Experiment Builder (Anwyl-Irvine et al., 2020), restricted to the Google Chrome web browser on a desktop or laptop computer, but with no operating system restrictions. Participants listened with their own wired headphones. Following informed consent, participants underwent an online headphone placement screening utilizing dichotic Huggins pitch to ensure compliance with the experiment’s headphone requirements (Milne et al., 2020). Here, a faint pitch can be detected in noise only when stimuli are perceived dichotically. Participants who failed to pass this headphone check did not progress to the main experiment. Due to a high headphone check failure rate during initial recruitment, a “second chance” headphone check was implemented to allow participants to correct their headphones and try again to pass the check. Participants who failed a second headphone check were rejected from the main experiment (note that this check is not designed to measure spatial hearing, but rather to assure proper headphone use). Additionally, each participant completed the English language version of the LexTALE questionnaire (Lemhöfer & Broersma, 2012) immediately after the main experiment. The LexTALE uses people’s familiarity with real words and phonotactic nonwords as a proxy measure of vocabulary and overall language proficiency.

In the main task, acoustic stimuli were presented randomly without replacement with visual presentation of a fixation cross. Following the stimulus presentation, a 6 (number) × 4 (color) grid with each color/number combination of the stimulus phrases appeared. Participants selected the color and number corresponding to those uttered in the target phrase using a trackpad or mouse click. Responses were scored as correct only if both the color and number selection were accurate. Traditionally, eight numbers are present in the CRM design. The number 7 was eliminated from the task design because of its bisyllabic pronunciation, and the number 8 was likewise eliminated for continuity.

Immediately prior to the main task, participants were instructed on the task procedure and completed five practice trials. Each practice stimulus presented an English distractor and the English target, Baron, at one of each of the five TMRs in random order. Following each practice trial, response feedback was provided in the form of an image of a green check mark or red cross appearing in the center of the screen to indicate correct or incorrect choices, respectively. Participants then completed 240 task trials without feedback. The trials were divided into ten blocks to accommodate the conditions of two distractor languages and five TMRs. Five blocks consisted of an English target with an English distractor; the remaining blocks consisted of an English target with a Mandarin distractor. The order of these five-block runs was counterbalanced across participants, and participants were notified when they had completed one set and were about to experience a switch in the language of the distractor phrase. Each block consisted of stimuli presented at one TMR; TMR presentation order was randomized within each distractor language condition. Optional 60-s breaks were provided between blocks. A progress bar accompanied the fixation screen to aid participant motivation. The time taken to complete the full task was approximately 12 min.

## Results

We analyzed recognition accuracy using a mixed model ANOVA, with masker language (Mandarin, English) and TMR (− 6 dB, − 3 dB, 0 dB, 3 dB, 6 dB) as a within-subject factor, and participant language proficiency (Mandarin, English) as a between-subject factor. (Note that we treat TMRs as categorical for analysis purposes, as the slope and linearity of the relationship between accuracy and step changes in TMR are not of theoretical importance here, e.g., the goal is not to establish the psychometric function for each condition and participant.) For both experiments, degrees of freedom for multi-level within-subjects variables were Greenhouse–Geisser corrected for violations of sphericity, and analyses were conducted in JASP 0.16.3 (JASP Team (2022). JASP, Version 0.16.3).

**Fig. 2:**
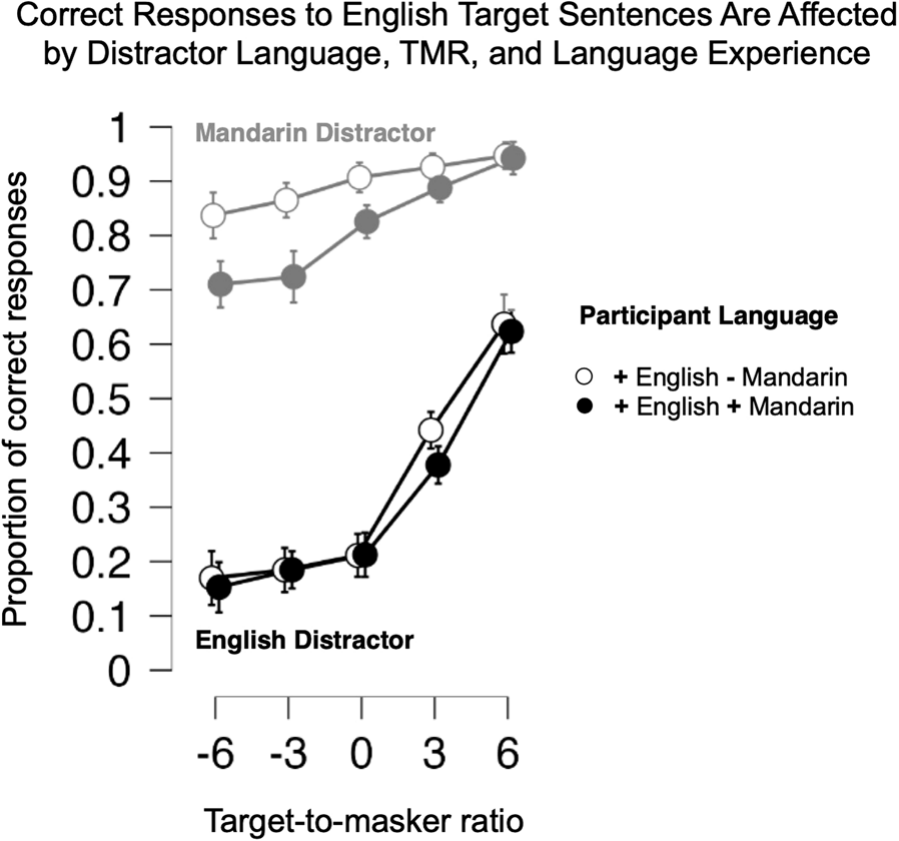
Experiment 1 Mean proportion correct trials (color/number responses) as a function of target-to-masker ratio (dB). All target sentences were spoken in English; responses with Mandarin-distractor masker shown in gray, and English-distractor masker in black. Responses from English speakers without access to Mandarin shown with hollow circles; responses from bilingual speakers with access to Mandarin shown with filled circles. Error bars show 95% confidence intervals. Chance response is 4%

As shown in Fig. 2, recognition of English-target sentences was considerably less accurate in the presence of English compared to Mandarin-distractor sentences [main effect of distractor language: F(1,56) = 1560.822, *p* < 0.0001, *η*^2^ = 0.672] and was overall adversely affected by lower TMR [main effect of TMR: F(2.80, 156.785) = 264.259, *p* =  < 0.0001, *η*^2^ = 0.134]. Mandarin listeners were overall slightly less accurate [main effect of F(1,56) = 4.610, *p* = 0.036, *η*^2^ = 0.005], an effect primarily driven by poorer performance under Mandarin distractors [interaction of language experience with masker language: F(1, 156.785) = 4.438, *p* = 0.032, *η*^2^ = 0.002]. English- and Mandarin-speaking participants were not differentially affected by TMR level changes [interaction of participants’ language with TMR: F(2.80, 156.79) = 2.458, *p* = 0.069, *η*^2^ = 0.001], but TMR effects differed according to whether English or Mandarin was the distractor language, with lower TMRs evoking much lower accuracy with English distractors than with Mandarin ones [interaction of distractor language with TMR: F(3.317, 185.731) = 69.436, *p* < 0.0001, *η*^2^ = 0.036].

As can be seen in Fig. 2, the two groups diverged in their susceptibility to more difficult listening conditions with Mandarin but not English distractors [3-way interaction of distractor language, TMR, and language experience: F(3.317, 185.731) = 4.955, *p* = 0.002, *η*^2^ = 0.003]. Mandarin–English bilinguals and English speakers did not perform significantly differently across English-distractor levels [sub-ANOVA of TMR x participant language, English distractors only: F(2.922, 163.644) = 1.019, *p* = 0.385, *η*^2^ = 0.003]. For English distractors, both groups showed overlapping “hockey stick”-like accuracy profiles, with near-chance performance with TMRs at 0 dB and below, and dramatic increases in accuracy from 0 to 3 dB and 3 to 6 dB TMRs.

By contrast, the intelligible Mandarin distractors led bilingual Mandarin–English participants to perform less accurately in reporting English targets for more difficult TMRs than participants who did not speak Mandarin, for whom the distractors were unintelligible [sub-ANOVA of TMR x participant language, Mandarin distractors only: F(3.10, 173.592) = 8.602, *p* < 0.0001, *η*^2^ = 0.028]. For all but the most advantageous TMR (+ 6 dB), Mandarin distractors significantly impeded Mandarin–English bilinguals’ English-target recognition relative to English speakers (Mann–Whitney post hoc tests, *p* < 0.05, corrected for 5 tests).

**Fig. 3:**
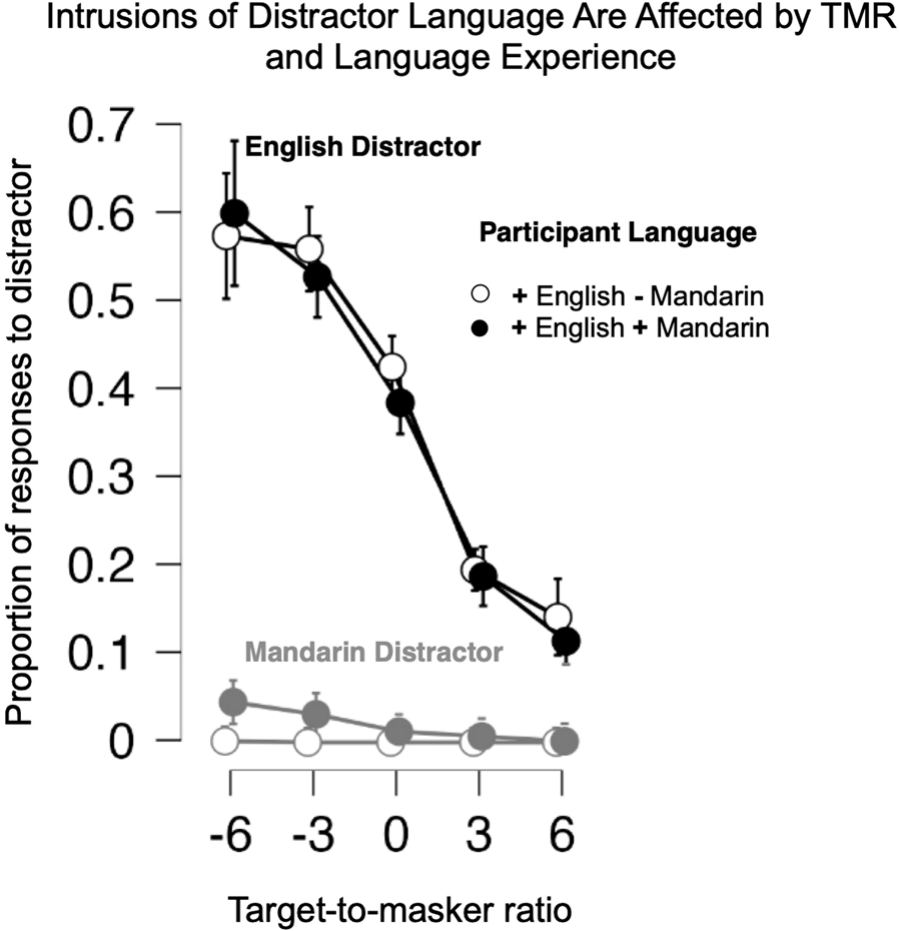
Experiment 1 Mean proportion *distractor* responses (intrusions) as a function of TMR (dB), distractor language, and language experience. Symbols and error bars as in Fig. 2

To better understand this pattern of results, we asked what proportion of the errors could be accounted for by intrusions from the distractor language, and how these patterns might differ across Mandarin–English bilinguals and English speakers (see Fig. 3). Intrusions were defined as color-number responses matching the masker phrase. To compare across TMRs, we divided the number of intrusions by the number of errors at each TMR for each distractor language. Because English speakers could not understand Mandarin and therefore had zero true intrusions (and only one coincidental instance across all participants), we analyzed data with English and Mandarin distractors separately, using ANOVA for English distractors, and Wilcoxon signed ranks for Mandarin distractors, for Mandarin-speaking bilinguals only.

As Fig. 3 shows, for English-distractor data, intrusion rate increased with decreasing TMR [main effect of TMR: F(2.191, 122.67) = 155.85, *p* < 0.0001, *η*^2^ = 0.633]. The majority of errors at challenging TMRs (where distractor amplitude > target amplitude) were intrusions from the distractor. At less challenging TMRs, such intrusions accounted for a minority of trials. This effect did not interact significantly with participants’ language [F(2.191, 122.67) = 0.645, *p* = 0.540, *η*^2^ = 0.003].

As shown in Fig. 3, a small proportion of Mandarin–English bilingual participants’ choices could be associated with the instruction from the distractor Mandarin sentence. Indeed, Wilcoxon signed-rank tests at each TMR level (uncorrected for multiple comparisons) showed a small proportion of bilingual Mandarin–English speakers’ errors in the − 6 dB (*V* = 120, *p* < 0.001), − 3 dB (*V* = 78, *p* = 0.002), and 0 dB (*V* = 28, *p* = 0.022) TMR conditions were attributable to the Mandarin-distractor instruction.

Finally, we examined how Mandarin speakers’ proficiency in English might modulate these experimental factors. A repeated-measures ANOVA (again Greenhouse–Geisser corrected for multi-level within-subjects factors) was run on bilingual Mandarin–English speakers only. The LexTALE measure of English proficiency was used as the continuous predictor variable, and distractor language and TMR were within-subjects factors, as in the previous analyses. LexTALE English proficiency (mean(SD) = 83.1(11.3), range 55.7–100) did not interact significantly with masker language [F(1, 28) = 0.073, *p* = 0.79, *η*^2^ < 0.001], but English proficiency significantly modulated the effect of the TMR [F(2.809, 78.660) = 6.795, *p* = 0.0056, *η*^2^ = 0.035]. Separate regressions of proficiency x recognition accuracy for each TMR level (collapsed over masker language) showed that English proficiency was strongly positively associated with recognition accuracy at 0 dB TMR (R^2^ = 0.42, *p* < 0.0001) and − 3 dB TMR (*R*^2^ = 0.27, *p* = 0.0036), moderately positively associated at − 6 dB and + 3 dB TMRs (*R*^2^ = 0.19, *p* = 0.0160 and *R*^2^ = 0.13, *p* < 0.0483, respectively), and not significantly associated with recognition accuracy at the least challenging + 6 TMR (*R*^2^ = 0.003, *p* = 0.786). This inverse-U-shaped interaction between proficiency and TMR was not significantly influenced by whether the masker was English or Mandarin [F(3.677, 102.942) = 0.50, *p* = 0.72].

In summary, when listening to English sentences, both English speakers’ and Mandarin–English bilinguals’ recognition was dramatically less accurate when the distracting talker also spoke in English, versus when he spoke in Mandarin. Indeed, for Mandarin–English bilinguals, recognition accuracy was 8.5% higher (pairwise contrast *p* < 0.001) with a − 6 dB TMR when Mandarin was the distractor than with a + *6 dB TMR* when English was a distractor. This advantage ballooned to 19.8% for English speakers (*p* < 0.00001). In other words, the TMR condition that should have rendered the target much easier to hear was actually quite difficult when the target and distractor languages matched. Likewise, the TMR condition that was expected to be the most challenging was actually made much easier when the target and distractor speech mismatched. Recall that this linguistic release from masking occurs even though the Mandarin- and English-distractor sentences are spoken by the same talker.

We also observe that Mandarin bilinguals’ English speech recognition is differentially affected by competing Mandarin speech, compared to their counterparts. This is particularly true at low TMRs, with a 12.5% (− 6 dB TMR) to 8% (0 dB TMR) decrement in performance. This finding is in line with previous research demonstrating an advantage in release from masking when the masker language is unfamiliar, and therefore unintelligible to the listener (e.g., Brouwer et al., 2012). This effect could be due to the increased effectiveness of a *salient* informational masker, such as a distracting speech cue providing conflicting task directions in a language accessible to the listener. Alternatively, it has been suggested that greater familiarity with a target speech stream in a masked auditory context may allow listeners to “tune in” by filtering irrelevant acoustic information (Van Engen, 2012). In the current experiment, the use of the same talker for the target and distractor languages in a speech task with restricted spectrotemporal variation minimized the ability of the listener to rely on talker-specific cues, preventing the listeners from “tuning in” to the target speech stream based on anything other than their prior experience with the linguistic content of the target and masker.

Regardless of distractor language, Mandarin–English bilinguals with greater estimated English proficiency were more successful in comprehending these semantically denuded English sentences when distractors were present, except at the most advantageous TMR. Finally, and rather strikingly, when English was both the target and masking language, decreasing TMR had statistically indistinguishable effects on English speakers and Mandarin–English bilinguals. In other words, the less perceptually salient acoustics of the target and masking signals at a lower TMR negated the recognition advantage that may be held by English speakers who do not understand Mandarin.

Two general questions are left unresolved by Experiment 1: (1) is bilinguals’ recognition in both their native language (Mandarin) and their fluent second language (English) similarly affected by different levels of distracting English or Mandarin speech?; (2) How is that effect modulated when the masker language matches or mismatches the attended target language? In Experiment 2, we again use our “one-man cocktail party” to ask whether participants’ sentence recognition is more robust in their native versus second language to different levels of distraction and “linguistic masking” when talker differences are minimized. Guided by the results of Experiment 1, we use the subset of TMRs (− 3, 0, + 3 dB) that evoked the greatest range of masking effects. This enables us to test all possible combinations of target and distractor language at each TMR level in a single online testing session that is not too lengthy or onerous for participants.

## Experiment 2

### Participants

As for Experiment 1, we used Prolific to screen and recruit 30 participants for online testing. One participant performed only at chance level for all Mandarin-target stimuli and was subsequently removed from analysis, resulting in a total sample size of 29 participants. All remaining participants (21 female, ages 18–35 inclusive) identified as native speakers of Mandarin Chinese with bilingual fluency in English in their Prolific intake survey, which we used for screening. All reported normal hearing. Appendix Tables 1, 2, and 3 share additional self-report language experience gathered from a demographic survey embedded in the experiment. The experiment procedures were approved by the Carnegie Mellon University Institutional Review Board.

### Stimuli

The stimuli were recorded using the same equipment and procedure as in Experiment 1. The bilingual fluency of Experiment 2 participants allowed for a full crossing of target and distractor language across English and Mandarin. Four conditions involved two language-matched stimulus sets—*English*_*target*_*English*_*distractor*_ and *Mandarin*_*target*_*Mandarin*_*distractor*_*—*and two language-mismatched stimulus sets—*English*_*target*_*Mandarin*_*distractor*_ and *Mandarin*_*target*_*English*_*distractor*_*.* The English-target stimuli from Experiment 1 were used along with a set of accompanying speech stimuli generated with a Mandarin target (see Table 1). These stimuli were created using recordings from the same speaker as in Experiment 1. “[tian kong]” was chosen as the Mandarin target and served as an analog to the English-target “Baron” when the target phrase was spoken in Mandarin. Call signs “Baron” and “[tian kong]” were never used simultaneously to minimize confusion and ensure the presence of a single target cue for each trial. 72 stimulus pairs were generated for each target–distractor language condition, resulting in a total of 288 stimulus pairs, presented over two experimental sessions on consecutive days. Each of three TMRs (− 3 dB, + 3 dB, and 0 dB, or equal in amplitude to the masker) was evenly distributed across the pool of stimuli, such that 96 stimuli were presented at each TMR level.

### Procedure

Figure 1 illustrates the approach to Experiment 2. Each participant completed each of the four conditions in counterbalanced order across two sessions, where both Mandarin- and English-target languages were presented in the context of a matched or mismatched distractor language. Participants completed two sessions, one for each target language. The order in which target languages were presented across sessions was counterbalanced. Each session lasted about 30 min. In each session, participants completed the headphone screening described above, a brief 5-trial practice session with feedback, and 144 trials of the main experimental task over 6 blocks, counterbalanced regarding distractor language and TMR, with optional 60-s breaks between blocks. Blocks were grouped such that the distractor language condition (English or Mandarin) was not interleaved; participants completed each block of trials consisting of one distractor language before continuing to the next. Within this grouping, TMR was presented by block in random order. Immediately following the first day’s CRM task, participants completed the LexTALE assessment of English proficiency. The second online session with the other target language was completed within 24 h. In blocks where Mandarin was the target language, Hanzi notation indicated the numbers (see Table 1); when English was the target, Arabic numerals indicated numbers in the response grid, as in Experiment 1.

## Results

We again used repeated-measures ANOVA, here with target language (Mandarin, English), masker language (Mandarin, English) and TMR (− 3 dB, 0 dB, 3 dB) as within-subject effects. As previously, Greenhouse–Geisser correction was applied for TMR main effects and interactions.

**Fig. 4:**
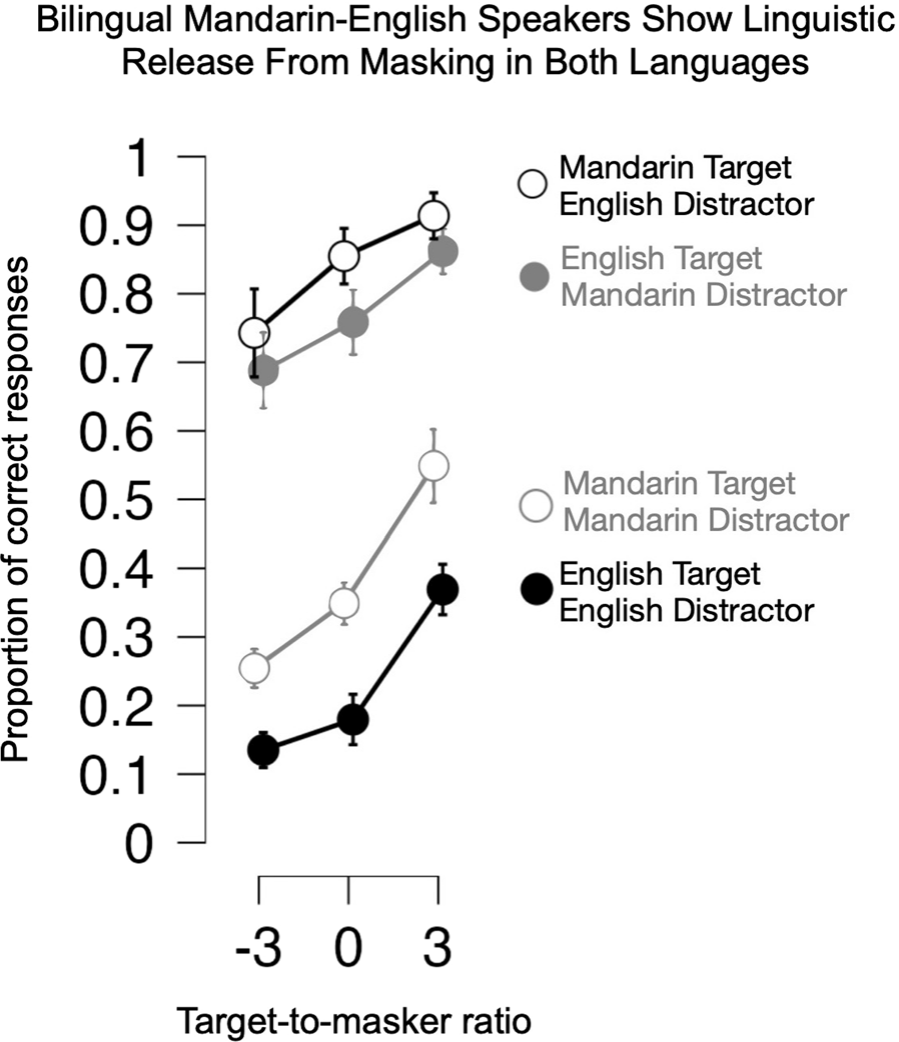
Experiment 2 Mean proportion correct responses as a function of TMR (in dB, x-axis), distractor language (Mandarin in gray shading, English in black shading), and target language (Mandarin in open circles, English in filled circles)

Mandarin–English bilingual participants were overall more accurate in comprehending targets in Mandarin compared to English [F(1, 28) = 24.759, *p* < 0.001, *η*^2^ = 0.037; they were also more accurate overall when distractors were Mandarin, versus English [F(1, 28) = 10.657, *p* = 0.003, *η*^2^ = 0.006]. Average accuracy increased with TMR [F(1.838, 51.462) = 172.023, *p* < 0.001, *η*^2^ = 0.095], but as can be seen in the roughly parallel lines in Fig. 4, there was no differential effect of TMR with target language [F(1.786, 50.005) = 2.338, *p* = 0.112] or distractor language [F(1.554, 43.509) = 1.390, *p* = 0.257].

Recognition accuracy was much higher when target and masker language mismatched versus matched [Interaction of target and masker language, F(1, 28) = 1085.577, *p* < 0.001, *η*^2^ = 0.725]. This was true for both Mandarin targets (45.3% difference) and English targets (54.2% difference). A post hoc sub-ANOVA comparing accuracy for Mandarin targets with English distractors versus English targets with Mandarin distractors showed no significant overall difference (F(1,28) = 4.155, *p* = 0.051), or interaction with TMR (F(1.493, 41.799) = 1.483, *p* = 0.238). By contrast, when target and masker language matched, accuracy for Mandarin speech (overall 38.4%) was significantly greater than for English (22.8%) (F(1,28) = 86.715, *p* < 0.001, *η*^2^ = 0.240); this accuracy advantage for Mandarin held across TMR levels (no significant interaction of distractor language with TMR, F(1.937, 54.244) = 2.247, *p* = 0.117, *η*^2^ = 0.007).

The three-way interaction of TMR, target language, and distractor language was significant [F(1.946, 54.491) = 15.328, *p* < 0.001, *η*^2^ = 0.007], albeit with a small effect size. Post hoc tests revealed no significant interaction with target or distractor language when TMR changed from -3 dB to 0 dB [F(1,28) = 1.043, *p* = 0.316, *η*^2^ < 0.01]. However, target and distractor language interacted with TMR at levels 0 dB and 3 dB [F(1,28) = 30.312, *p* < 0.001, *η*^2^ = 0.010]; as can be seen in Fig. 4, there was greater improvement in accuracy from 0 to 3 dB when target and distractor language matched versus when they mismatched. To describe the effect slightly differently, when target and distractor language mismatched, the increases in accuracy from − 3 to 0 and 0 to 3 TMRs were similar. However, when target and distractor language matched, there was a disproportionate gain in accuracy between 0 and 3 dB TMR, compared to − 3 dB to 0 TMR.

**Fig. 5:**
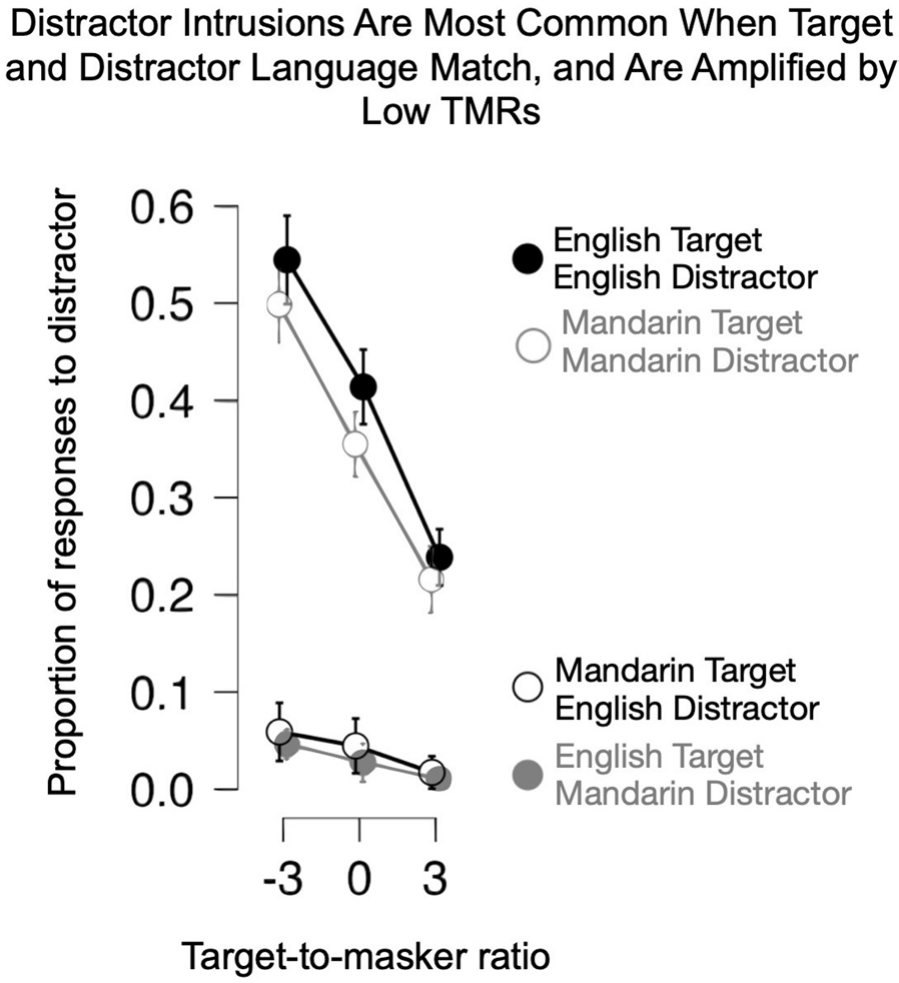
Experiment 2 Mean proportion distractor responses (intrusions) as a function of TMR (in dB, x-axis), distractor language (Mandarin distractor in gray shading, English distractor in black shading), and target language (open circle Mandarin, filled circles English)

As in Experiment 1, we asked what proportion of errors could be accounted for by intrusions from the distractor talker, and how this proportion might vary with target and distractor language along with TMR. As shown in Fig. 5, there were few intrusions in target–distractor language-mismatched conditions (near floor), compared to a high proportion of intrusions in target–distractor language-matched conditions. Thus, target–distractor match and mismatch conditions were analyzed separately, using non-parametric tests for mismatch conditions.

For conditions when target and distractor languages matched, a repeated-measures ANOVA showed a very large overall TMR effect on proportion of distractor intrusions (F(1.63, 45.562) = 121.816, *p* < 0.001), with decreasing proportion of errors with higher TMR, and a slightly (4.1%) overall higher proportion of intrusions for English versus Mandarin (F(1, 28) = 5.966, *p* = 0.021). This difference did not change significantly with TMR (F(1.910, 53.470) = 0.681, *p* = 0.504). For target–distractor language-mismatching conditions, Wilcoxon signed ranks tests showed no significant differences in proportion of intrusions between English and Mandarin targets with language-mismatched distractors at any TMR level (all *p* > 0.1).

Finally, as with Experiment 1, we asked whether LexTALE-assessed English language proficiency modulated the observed effects. LexTALE scores for bilingual Mandarin–English speakers (mean(SD) = 77.45(15.8), range 47.5–100) were numerically but not significantly lower in Experiment 2 compared to Expt 1, t(57) = 2, *p* = 0.1)).

In contrast to the Experiment 1 results and our predictions, we found no significant interaction of English proficiency with any main effect or interaction (all *p* > 0.2); this held true when we limited the analysis to only English targets (all *p* > 0.2), or only English distractors (all *p* > 0.2).

## General discussion

Listening to speech in the context of distracting background talkers requires selective attention to segregate and select information from the mix of sounds reaching the ears. This is the crux of the cocktail party challenge, which is exacerbated by age, hearing and communication disorders, and listening in a non-native language. Here, we created a “one-man bilingual cocktail party” to minimize differences in salient low-level acoustic cues that help to distinguish talkers such as voice quality and vocal tract length. We relied on a task that utilized closed-set recognition of high-frequency, familiar words and eliminated differential syntactic and semantic cues that could support recognition in the context of distracting speech. Further, we time-aligned targets and distractors to minimize “acoustic glimpses” of the target within brief silences in the distractor speech. These conditions resulted in very high selective attention demands and established a context (albeit an artificial one) in which to examine linguistic release from masking among English listeners (with no Mandarin proficiency) versus bilingual Mandarin–English listeners.

Results from both Experiment 1 and Experiment 2 conformed with the previously described *linguistic release from masking* phenomenon, whereby speech is more difficult to recognize with a distractor from the same language, compared to a different language. Noun recognition is of course not the only prerequisite to fluency in a language, but as recognition necessarily comes before comprehension, it is a useful metric for approximating a listener’s attunement to a language as well as the attentional effects that the language imparts. In Experiment 1, regardless of their language experience, participants performed substantially more poorly when targets and distractors were both in English than when the target was in English, but the distractor was in Mandarin. This influence was considerable: recognition improved by 53.5% on average with a mismatched, compared to a matched, language distractor. Moreover, the advantage was evident both when the distractor was intelligible (bilingual Mandarin–English listeners) and also when it was unintelligible (English listeners without access to Mandarin). Among Experiment 2 Mandarin–English bilinguals with self-reported native Mandarin experience, linguistic release from masking was apparent in both languages. When attending to Mandarin, having English as the distractor improved performance 45.3% relative when Mandarin was the distractor; when attending to English, the release from masking was even larger, with a 54.2% improvement when Mandarin was the distractor versus English. In other words, the linguistic release from masking was even greater to the *non-native* language.

Language experience overall had a lesser influence. The language experience advantage for comprehending English targets was small in Experiment 1: overall, the difference in accuracy between English speaking participants without access to Mandarin compared to Mandarin–English bilinguals was less than 5%. It is worth noting that CRM is a closed-set task with a consistent structure across many trials. In real-world speech contexts with greater variability, it is reasonable to expect a somewhat greater difference in accuracy. Moreover, with stimulus materials that minimized language- and culturally specific information, bilingual Mandarin–English listeners experienced a very similar degree of interference from English distractors across TMR levels, relative to English listeners who had no access to Mandarin. By contrast, when Mandarin speech was the distractor, participants’ language experience (and thus Mandarin-distractor intelligibility) influenced accuracy when demands on selective attention were the greatest, with bilingual English-Mandarin listeners differentially more affected by Mandarin distractors at the more challenging TMRs. In these conditions, Mandarin-distractor keywords significantly intruded in Mandarin listeners’ responses to English targets. Finally, in Experiment 2, there was a modest influence of language experience, with bilingual participants who had Mandarin as their self-reported L1 but were also proficient in English exhibiting a 11.2% advantage in comprehending Mandarin targets compared to English targets. Recognition also was overall more accurate (4.4%) with Mandarin, versus English distractors.

These findings are in agreement with previous research on linguistic release in masking. In an experiment quite similar to our Experiment 1, Van Engen (2010) examined speech-in-babble recognition in native English and native Mandarin listeners attending to English-target speech with English or Mandarin two-talker babble. The findings of the present study largely mirrored that publication: Speech-in-noise performance increased for all participants concomitant with increasing SNR, speech-in-noise recognition for English targets was lower in English babble than Mandarin babble for all participants, and native Mandarin listeners demonstrated significantly worse speech-in-noise performance for English targets with Mandarin babble than native English listeners. In sum, when the target and masker signals are highly similar (e.g., the same language), listeners experience increased difficulty when parsing the target speech from the masking context. However, English speakers without access to Mandarin experienced greater linguistic release from informational masking in Mandarin babble than did Mandarin speakers. Our use of the same speaker for the target and masking speech provides further evidence for these effects existing primarily at the linguistic level rather than resulting from talker-specific acoustic characteristics. Furthermore, the closed-set format of CRM allows for the calculation of masker *intrusions* on speech recognition. In Experiment 1, we found that responses made in favor of the distracting Mandarin speech stream were above chance for Mandarin listeners, but not for English listeners without access to Mandarin. This indicates the influence of language experience specific to informational masking as opposed to energetic masking, which did not vary between listeners.

In Experiment 2, speech recognition performance was always worse for matched target and masker speech cue conditions than in mismatched language conditions, further corroborating the speech recognition benefit that may be gained from distinguishable and accessible linguistic content in the target speech stream. Notably, Van Engen (2010) utilized two-talker babble, which increases energetic masking relative to the target, whereas our version of CRM utilized a single masking speaker. Further research is needed to determine the effect of energetic masking in the form of speech-shaped babble and environmental noise on linguistic release from masking to improve our understanding of this effect in varied and ecologically valid contexts.

A few questions remain outstanding. For instance, two implicit assumptions made in the present study are that Mandarin speakers who are unfamiliar with English will (1) show similar levels of linguistic release from masking in Mandarin-target/English-distractor conditions as the Experiment 1 English speakers did in the English-target/Mandarin-distractor condition and (2) show similar masking by Mandarin targets compared to English non-Mandarin speakers with English targets. Further studies will be needed to assess this, particularly given the counterintuitive yet robust findings of *reversed* linguistic release from masking with particular talker combinations, e.g., with gender-mismatched talkers (Williams & Viswanathan, 2020). Another is the degree to which language experience and ability might benefit performance with more natural spoken language stimuli. The CRM is an impoverished model of language, in that it requires only the most basic color and number vocabulary to be incorporated in a single rudimentary syntactic structure. Further investigation is needed to determine the effect of linguistic release from masking in more natural speech conditions.

Participants who registered for the study using the online recruitment platform Prolific were required to self-report their language history, including native language and other learned languages. This served as the basis for screening in our recruitment of “native English” and “Mandarin–English bilingual” online participants. As a verification step, we also included a brief language experience survey in the experiment (therefore following the Prolific recruitment screening). Interestingly, participants’ responses sometimes differed across these two assays of language experience. In our analyses, we used the Prolific intake survey to define our groups. Acknowledging the discrepancies in several participants’ self-report, two aspects of our study make us confident that our groups conform to expectations. First, Mandarin–English bilinguals needed to perform the task in each language to succeed (only 1 participant, excluded from analyses, failed to do so). Second, instructions for segments of Experiment 2 involving Mandarin targets were presented in Mandarin thereby demanding familiarity with written Mandarin. Third, all participants completed the LexTALE test providing us a quantitative metric of English proficiency. Even so, the discrepancy in self-reported language background across testing time points is a caution for future studies: participants can be unreliable reporters. Further research with bilingual populations, especially that which takes place online, ought to emphasize consistency in reporting of language experience and include additional information such as the ages at which languages were learned, and the extent of their use through the participants’ lifetimes. Greater understanding of participants’ language history and the demands placed on their language use will further inform the effects of bilingualism on auditory processing (von Hapsburg & Peña, 2002).

The stimuli developed for the present studies are novel in presenting a bilingual cocktail party challenge that mitigates low-level voice identity cues that support selective attention. All stimulus materials are available via the Open Science Framework, (https://osf.io/spnfh/) in anticipation that they will be valuable in promoting research and advancing effective clinical and educational interventions among an ever-growing population of Mandarin–English bilingual listeners.

In summary, our introduction of a “one-man cocktail party” across highly distinct languages helps to tease apart contributions to informational masking. In normal cocktail party listening environments with multiple talkers, vocal characteristics unique to a speaker may be used for location and identification of an intended target. By using a single speaker with high fluency in both English and Mandarin and a closed-set CRM task with competing utterances well aligned in time, we established a unique selective attention challenge less supported by bottom-up voice differences, rhythm across utterances, or top-down linguistic knowledge about patterns of syntax or semantics. In all, the “release” from masking in this one-man bilingual cocktail party appears to have been driven predominantly by lower-level auditory features that differentiate Mandarin and English, with more subtle effects of higher-order language processing and expertise that emerge. These effects seem to exist across all but the least challenging listening condition, but are made most apparent in more demanding listening contexts.

## Data Availability

The data are openly available at https://osf.io/spnfh/. The experiments were not preregistered.

## Appendix

See Tables

**Table 2:** Participants in the “Native English” group in Experiment 1, as screened through their Prolific profile by a “monolingual English” self-report also provided additional demographic information after performing Experiment 1: age (in years), gender, ethnicity, race, native language(s), and other learned language(s)

Subject	Age (y)	Gender	Ethnicity	Race	Country of Birth*	Country of Residence*	Native Language(s)	Other Language(s)
1_1	23	Male	Not Hispanic or Latino	White	UK	UK	English	None
#1_2	23	Prefer Not to Answer	Not Hispanic or Latino	Black or African American	South Africa	South Africa	Zulu	Zulu and Sotho
1_3	32	Female	Not Hispanic or Latino	White	UK	UK	English	None
1_4	28	Male	Not Hispanic or Latino	White	UK	UK	English	None
# 1_5	29	Male	Not Hispanic or Latino	Black or African American	South Africa	South Africa	IsiZulu, English	IsiXhosa
1_6	24	Male	Not Hispanic or Latino	White	UK	UK	English	None
☨ 1_7	31	Male	Not Hispanic or Latino	Asian	USA	USA	English, Chinese	Cantonese
1_8	22	Female	Not Hispanic or Latino	White	UK	UK	English	None
1_9	20	Male	Not Hispanic or Latino	Black or African American	Canada	Canada	English	None
1_10	25	Male	Not Hispanic or Latino	Asian	Canada	Canada	English	None
1_11	21	Female	Not Hispanic or Latino	Black or African American	USA	USA	English	None
1_12	26	Female	Not Hispanic or Latino	White	Canada	Canada	English	French
1_13	21	Female	Hispanic or Latino	More than One Race	USA	USA	English	None
1_14	29	Female	Not Hispanic or Latino	White	USA	USA	English	None
1_15	31	Male	Not Hispanic or Latino	White	USA	USA	English	None
1_16	27	Male	Not Hispanic or Latino	White	Canada	Canada	English	French
1_17	29	Female	Not Hispanic or Latino	Asian	Japan	USA	English	Japanese
1_18	32	Male	Not Hispanic or Latino	White	UK	UK	English	None
1_19	26	Female	Not Hispanic or Latino	White	UK	UK	English	Spanish
1_20	34	Female	Not Hispanic or Latino	White	UK	UK	English	None
1_21	24	Male	Not Hispanic or Latino	Asian	N/A	N/A	Thai	English
1_22	29	Male	Not Hispanic or Latino	White	Australia	Australia	English	None
☨ 1_23	26	Female	Not Hispanic or Latino	Asian	Malaysia	UK	English	Cantonese, Malay, Mandarin Chinese
1_24	27	Male	Not Hispanic or Latino	White	USA	USA	English	None
1_25	26	Female	Not Hispanic or Latino	Asian	USA	USA	English	None
1_26	29	Male	Hispanic or Latino	White	USA	USA	English	None
1_27	19	Male	Not Hispanic or Latino	Prefer Not to Answer	Australia	Australia	English	None
1_28	30	Female	Not Hispanic or Latino	Asian	Canada	Canada	English	None
1_29	33	Female	Not Hispanic or Latino	White	UK	UK	English	None
1_30	26	Male	Not Hispanic or Latino	Asian	India	Spain	English	English, Tamil, Kannada, Malayalam, Hindi

The Experiment 1 demographic survey defined native language(s) as “any language learned before the age of 2.” ☨ Participants who self-reported knowledge of Chinese, regardless of dialect, were excluded from the study. * Country of birth and residence are collected by Prolific. # Two participants did not report English as a native or other language in our survey, but self-reported native English to Prolific and completed Experiment 1 in English

2,

**Table 3:** Participants in the “Native Mandarin” group in Experiment 1, as screened through their Prolific profile by a “native Mandarin” self-report also provided additional demographic information after performing Experiment 1: age (in years), gender, ethnicity, race, country of birth, country of residence, native language(s), and other learned language(s)

Subject	Age (y)	Gender	Ethnicity	Race	Country of Birth*	Country of Residence*	Native Language(s)	Other Language(s)
1_31	20	Female	Not Hispanic or Latino	Asian	Hungary	UK	Hungarian, Chinese	English
1_32	20	Male	Not Hispanic or Latino	Asian	Canada	Canada	Cantonese	Chinese
1_33	23	Female	Not Hispanic or Latino	Asian	China	Canada	English, Mandarin	None
1_34	19	Male	Not Hispanic or Latino	Asian	China	UK	Chinese (Mandarin)	English
1_35	26	Male	Not Hispanic or Latino	Asian	N/A	N/A	Chinese	French, English
1_36	23	Male	Not Hispanic or Latino	More than One Race	Portugal	Portugal	Chinese	English, Portuguese
1_37	22	Non-Binary	Not Hispanic or Latino	Asian	China	Canada	Mandarin, Cantonese	English
1_38	23	Male	Not Hispanic or Latino	Asian	Canada	Canada	English, Chinese	None
1_39	20	Female	Not Hispanic or Latino	Asian	China	Canada	Chinese, English	None
1_40	26	Male	Not Hispanic or Latino	Asian	Hong Kong	Canada	Cantonese, English	Cantonese
1_41	22	Male	Not Hispanic or Latino	Asian	Malaysia	UK	Chinese, Malay, English	Chinese
1_42	26	Male	Not Hispanic or Latino	Asian	China	Canada	English, Mandarin	Mandarin
1_43	24	Male	Not Hispanic or Latino	Asian	China	Canada	Mandarin	English
1_44	26	Female	Not Hispanic or Latino	Asian	N/A	N/A	Chinese (Mandarin)	English
1_45	22	Male	Not Hispanic or Latino	Asian	China	Canada	Cantonese	English
1_46	32	Male	Not Hispanic or Latino	Asian	China	Canada	Chinese (Mandarin)	English
1_47	25	Male	Prefer Not to Answer	Asian	Malaysia	UK	English, Mandarin	Mandarin
1_48	28	Female	Not Hispanic or Latino	Asian	Canada	Canada	English, Chinese	None
1_49	35	Male	Not Hispanic or Latino	Asian	China	Canada	Chinese	English
1_50	25	Female	Not Hispanic or Latino	Asian	Taiwan	Australia	Mandarin	Mandarin
1_51	28	Female	Not Hispanic or Latino	Asian	China	Canada	Chinese	Cantonese Chinese
1_52	20	Female	Not Hispanic or Latino	Asian	Canada	Canada	Cantonese	Cantonese
1_53	23	Female	Not Hispanic or Latino	Asian	China	Canada	Mandarin	English
1_54	23	Female	Not Hispanic or Latino	Asian	China	Canada	Mandarin, Cantonese	English
1_55	20	Female	Not Hispanic or Latino	Asian	China	Canada	Chinese (Mandarin)	English
1_56	19	Female	Not Hispanic or Latino	Asian	China	Canada	English	Chinese
1_57	31	Female	Prefer Not to Answer	Asian	Hong Kong	Canada	English, Cantonese	Spanish
1_58	21	Female	Not Hispanic or Latino	Asian	Canada	Canada	English, Mandarin	Cantonese
1_59	23	Female	Not Hispanic or Latino	Asian	Canada	Canada	English, Chinese	English, Chinese
1_60	33	Male	Not Hispanic or Latino	Asian	China	USA	English	Chinese

The Experiment 1 demographic survey defined native language(s) as “any language learned before the age of 2.” * Country of birth and residence are collected by Prolific

3 and

**Table 4:** Participants who took part in Experiment 2, as screened through their Prolific profile by a “native Mandarin” self-report with bilingual proficiency in English, also provided additional demographic information after performing Experiment 1: age (in years), gender, ethnicity, race, country of birth, country of residence, native language(s), and other learned language(s)

Subject	Age (y)	Gender	Ethnicity	Race	Country of Birth*	Country of Residence*	Native Language(s)	Other Language(s)
2_1	21	Female	Not Hispanic or Latino	Asian	USA	USA	Mandarin, English	None
2_2	21	Male	Not Hispanic or Latino	Asian	Canada	Canada	English, Cantonese	French
2_3	29	Female	Not Hispanic or Latino	Asian	China	Canada	Chinese	Chinese, English
2_4	30	Male	Not Hispanic or Latino	Asian	China	USA	Mandarin	English
2_5	26	Male	Not Hispanic or Latino	Asian	Canada	Canada	English, Chinese	Chinese
☨ 2_6	23	Male	Not Hispanic or Latino	Asian	Hong Kong	USA	Cantonese	English
2_7	19	Female	Not Hispanic or Latino	Asian	USA	USA	English, Chinese	None
2_8	24	Female	Not Hispanic or Latino	Asian	Hong Kong	UK	English	Chinese
2_9	20	Male	Not Hispanic or Latino	Asian	China	Spain	Chinese	English, Spanish
2_10	23	Female	Not Hispanic or Latino	Asian	Denmark	UK	Chinese, English	Russian
2_11	18	Male	Not Hispanic or Latino	Asian	Germany	Germany	Chinese, German	English
2_12	24	Female	Prefer Not to Answer	Asian	China	UK	Chinese	English
2_13	25	Female	Not Hispanic or Latino	Asian	Taiwan	USA	Chinese	English
2_14	24	Female	Not Hispanic or Latino	Asian	China	Netherlands	Chinese, English	None
2_15	19	Female	Not Hispanic or Latino	Asian	USA	USA	Mandarin	English
2_16	21	Female	Not Hispanic or Latino	Asian	China	UK	Mandarin	English
2_17	34	Female	Not Hispanic or Latino	Asian	Hong Kong	Canada	English, Cantonese	Cantonese
2_18	35	Female	Not Hispanic or Latino	Asian	USA	USA	Chinese	English
2_19	21	Female	Not Hispanic or Latino	Asian	USA	USA	English, Cantonese	None
2_20	25	Male	Not Hispanic or Latino	Asian	Taiwan	USA	Mandarin, Taiwanese	Mandarin
2_21	20	Male	Not Hispanic or Latino	Asian	Canada	Canada	Cantonese, Mandarin	English
2_22	24	Female	Not Hispanic or Latino	Asian	USA	USA	English, Chinese	None
2_23	27	Female	Not Hispanic or Latino	Asian	USA	USA	Chinese, English	Chinese, English
2_24	24	Female	Not Hispanic or Latino	Asian	Taiwan	Canada	English, Chinese (Mandarin)	Chinese (Mandarin)
2_25	18	Female	Not Hispanic or Latino	Asian	Canada	Canada	Mandarin, English	None
2_26	31	Male	Not Hispanic or Latino	Asian	China	Canada	Chinese	Chinese, English
2_27	20	Female	Not Hispanic or Latino	Asian	China	Canada	Chinese	English
2_28	18	Female	Not Hispanic or Latino	Asian	USA	USA	English, Chinese	None
2_29	31	Female	Not Hispanic or Latino	Asian	UK	UK	Mandarin	English, Japanese
2_30	20	Female	Not Hispanic or Latino	Asian	USA	USA	Chinese	English

The Experiment 2 demographic survey defined native language(s) as “any language learned before the age of 2.” ☨ One participant who self-reported fluency in Mandarin indicated a lack of keyword recognition by poor task performance for the Mandarin target, with superior task performance for the English target, and was thus removed from analysis. * Country of birth and residence are collected by Prolific

4

## Abbreviations

CRM: Coordinate response measure
TMR: Target-to-masker ratio
